# Supplementary material for: Unravelling agronomic performance and genetic diversity of newly developed maize inbred lines for arid conditions
Source: PeerJ. 2025 Jun 27;13:e19598. doi: 10.7717/peerj.19598 (PMC12208108; doi:10.7717/peerj.19598)
Supplement: Supplemental Information 1 [file peerj-13-19598-s001.docx]

Table S1. Code, name, origin and pedigree of the fourteen maize inbred lines used in this study

| **Name** | **Origin** | **Pedigree** |
| --- | --- | --- |
| LA442B | Nigeria | Tux PoolC2lC2-5 |
| LCA332 | Ethiopia | {POOL 9 Ac3-SR(BC2) FS2-6-2-3 ENT40 |
| LMP214A | India | POOL 9Ac7-SR(BC2) FS123-1-2-2ENT 25 |
| LZAm7B | Zambia | Pool15QPMFS309-B-1-B-B-B |
| ZBm40A | Zambia | ZEWAc1F2-204-2-1-B-1 |
| SNY23 | Mexico | Mexico Acc No. 3136 |
| RA28C | Ethiopia | Pop 30-145-1-1 # 1-2-1-2 |
| B17AB | India | HKI 325-17AF1 |
| DKC14 | India | Pool 28, sub-tropical |
| DKCA2 | India | Pop 147-F2#102-1-1 |
| IKA22 | Mexico | CML 31 POB 27 C5 |
| SSK36 | Mexico | IPA 3-20-1-1-1-2 |
| LZP210 | Mexico | CML 143P62 C6HC88 |
| LCM54 | Mexico | CML 222 CL36 |
