# Supplementary material for: Unravelling agronomic performance and genetic diversity of newly developed maize inbred lines for arid conditions
Source: PeerJ. 2025 Jun 27;13:e19598. doi: 10.7717/peerj.19598 (PMC12208108; doi:10.7717/peerj.19598)
Supplement: Supplemental Information 2 [file peerj-13-19598-s002.docx]

**Table S2.** Monthly minimum temperature (MinT), maximum temperature (MaxT), growing degree day (G.D.D), relative humidity (RH), and precipitation (Perc) during seasons of 2021 to 2023 alongside 40-year averages (1983–2023).

| **Mon.** | **MinT (° C)** | **MaxT (° C)** | **G.D.D* (° C)** | **RH (%)** | **Perc (mm)** |
| --- | --- | --- | --- | --- | --- |
|  | **First season (2021)** | | | | |
| May | 20.08 | 34.68 | 430 | 63.37 | 0.0 |
| June | 22.11 | 35.48 | 459 | 65.85 | 0.0 |
| July | 25.14 | 38.18 | 563 | 66.90 | 0.0 |
| August | 26.12 | 39.45 | 598 | 67.30 | 0.0 |
| September | 24.75 | 37.01 | 521 | 64.95 | 0.0 |
|  | **Second season (2022)** | | | | |
| May | 18.85 | 32.94 | 384 | 69.51 | 0.00 |
| June | 23.10 | 36.41 | 488 | 67.43 | 0.00 |
| July | 24.61 | 37.81 | 549 | 66.48 | 0.21 |
| August | 25.77 | 38.19 | 573 | 66.42 | 0.01 |
| September | 24.89 | 37.45 | 530 | 64.72 | 0.09 |
|  | **Third season (2023)** | | | | |
| May | 17.96 | 32.83 | 378 | 66.64 | 0.72 |
| June | 22.39 | 36.04 | 472 | 67.81 | 1.36 |
| July | 24.47 | 39.28 | 585 | 66.60 | 0.00 |
| August | 25.90 | 38.43 | 579 | 66.98 | 0.00 |
| September | 25.98 | 38.94 | 569 | 64.48 | 0.89 |
|  | **40 years average (1983-2023)** | | | | |
| May | 18.75 | 31.00 | 381 | 65.53 | 0.95 |
| June | 21.94 | 33.77 | 461 | 66.32 | 0.28 |
| July | 23.99 | 35.41 | 533 | 67.86 | 0.17 |
| August | 24.83 | 35.83 | 553 | 67.95 | 0.01 |
| September | 23.89 | 34.51 | 501 | 66.27 | 0.25 |

* Growing degree days were estimated using daily maximum and minimum temperatures, with a base temperature of 10°C, and accumulated for each month.
