# Supplementary material for: Unravelling agronomic performance and genetic diversity of newly developed maize inbred lines for arid conditions
Source: PeerJ. 2025 Jun 27;13:e19598. doi: 10.7717/peerj.19598 (PMC12208108; doi:10.7717/peerj.19598)
Supplement: Supplemental Information 3 [file peerj-13-19598-s003.docx]

**Table S3.** Genetic distance among 14 Egyptian maize inbred lines based on SCoT markers data.

| **Genotype** | LA442B | LCA332 | LMP214A | LZAm7B | ZBm40A | SNY23 | RA28C | B17AB | DKC14 | DKCA2 | IKA22 | SSK36 | LZP210 | LCM54 |
| --- | --- | --- | --- | --- | --- | --- | --- | --- | --- | --- | --- | --- | --- | --- |
| LA442B | 0.00 |  |  |  |  |  |  |  |  |  |  |  |  |  |
| LCA332 | 5.00 | 0.00 |  |  |  |  |  |  |  |  |  |  |  |  |
| LMP214A | 5.57 | 4.47 | 0.00 |  |  |  |  |  |  |  |  |  |  |  |
| LZAm7B | 4.58 | 4.47 | 4.24 | 0.00 |  |  |  |  |  |  |  |  |  |  |
| ZBm40A | 4.58 | 4.47 | 5.10 | **4.00** | 0.00 |  |  |  |  |  |  |  |  |  |
| SNY23 | 5.20 | 4.47 | 5.48 | 4.90 | 4.90 | 0.00 |  |  |  |  |  |  |  |  |
| RA28C | 5.20 | 4.69 | 4.47 | 4.47 | 4.90 | 5.10 | 0.00 |  |  |  |  |  |  |  |
| B17AB | 5.29 | 5.00 | 4.36 | 4.36 | 4.80 | 5.20 | 4.12 | 0.00 |  |  |  |  |  |  |
| DKC14 | 5.48 | 5.74 | 5.00 | 5.20 | 4.80 | 4.58 | 4.36 | 4.47 | 0.00 |  |  |  |  |  |
| DKCA2 | 6.08 | 6.32 | 6.16 | **6.48** | 5.83 | 5.83 | 5.48 | 5.39 | 4.80 | 0.00 |  |  |  |  |
| IKA22 | 5.48 | 5.57 | 5.57 | 5.57 | 5.57 | 5.74 | 4.80 | 4.47 | 5.29 | 5.74 | 0.00 |  |  |  |
| SSK36 | 5.39 | 5.10 | 5.29 | 5.10 | 4.69 | 4.90 | 4.69 | 4.58 | 4.80 | 6.00 | 4.36 | 0.00 |  |  |
| LZP210 | 5.29 | 5.00 | 5.20 | 5.00 | 5.00 | 4.80 | 5.20 | 4.47 | 5.29 | 5.57 | 4.90 | 4.80 | 0.00 |  |
| LCM54 | 4.90 | 5.39 | 5.57 | 4.80 | 4.80 | 4.58 | 5.00 | 4.47 | 5.10 | 5.57 | 4.47 | 4.80 | **4.00** | 0.00 |
