# Supplementary material for: Unravelling agronomic performance and genetic diversity of newly developed maize inbred lines for arid conditions
Source: PeerJ. 2025 Jun 27;13:e19598. doi: 10.7717/peerj.19598 (PMC12208108; doi:10.7717/peerj.19598)
Supplement: Supplemental Information 4 [file peerj-13-19598-s004.docx]

**Table S4.** Similarity coefficient (Dice measurement) among 14 Egyptian maize inbred lines based on SCoT markers data.

| **Genotype** | LA442B | LCA332 | LMP214A | LZAm7B | ZBm40A | SNY23 | RA28C | B17AB | DKC14 | DKCA2 | IKA22 | SSK36 | LZP210 | LCM54 |
| --- | --- | --- | --- | --- | --- | --- | --- | --- | --- | --- | --- | --- | --- | --- |
| LA442B | 1.00 |  |  |  |  |  |  |  |  |  |  |  |  |  |
| LCA332 | 0.77 | 1.00 |  |  |  |  |  |  |  |  |  |  |  |  |
| LMP214A | 0.71 | 0.81 | 1.00 |  |  |  |  |  |  |  |  |  |  |  |
| LZAm7B | 0.81 | 0.81 | 0.83 | 1.00 |  |  |  |  |  |  |  |  |  |  |
| ZBm40A | 0.82 | 0.82 | 0.76 | **0.86** | 1.00 |  |  |  |  |  |  |  |  |  |
| SNY23 | 0.74 | 0.81 | 0.70 | 0.77 | 0.78 | 1.00 |  |  |  |  |  |  |  |  |
| RA28C | 0.73 | 0.78 | 0.79 | 0.80 | 0.77 | 0.72 | 1.00 |  |  |  |  |  |  |  |
| B17AB | 0.73 | 0.75 | 0.80 | 0.81 | 0.78 | 0.72 | 0.81 | 1.000 |  |  |  |  |  |  |
| DKC14 | 0.71 | 0.68 | 0.74 | 0.73 | 0.78 | 0.78 | 0.79 | 0.792 | 1.00 |  |  |  |  |  |
| DKCA2 | 0.65 | 0.62 | 0.62 | **0.60** | 0.69 | 0.66 | 0.68 | 0.707 | 0.76 | 1.00 |  |  |  |  |
| IKA22 | 0.71 | 0.69 | 0.68 | 0.69 | 0.71 | 0.66 | 0.75 | 0.792 | 0.70 | 0.66 | 1.00 |  |  |  |
| SSK36 | 0.72 | 0.75 | 0.72 | 0.75 | 0.80 | 0.76 | 0.77 | 0.788 | 0.76 | 0.64 | 0.80 | 1.00 |  |  |
| LZP210 | 0.73 | 0.76 | 0.73 | 0.76 | 0.77 | 0.77 | 0.71 | 0.796 | 0.71 | 0.69 | 0.75 | 0.77 | 1.00 |  |
| LCM54 | 0.76 | 0.71 | 0.68 | 0.77 | 0.78 | 0.78 | 0.73 | 0.792 | 0.72 | 0.68 | 0.79 | 0.76 | 0.83 | 1.00 |
